# Association between subclinical thyroid dysfunction and depressive symptoms in the Korean adult population: The 2014 Korea National Health and Nutrition Examination Survey

**DOI:** 10.1371/journal.pone.0202258

**Published:** 2018-08-14

**Authors:** Jae Won Hong, Jung Hyun Noh, Dong-Jun Kim

**Affiliations:** Department of Internal Medicine, Ilsan-Paik Hospital, College of Medicine, Inje University, Koyang, Gyeonggi-do, Republic of Korea; National Institue on Drug Abuse, UNITED STATES

## Abstract

**Background:**

Clinical hyper and hypothyroidism are associated with a risk for depression.

**Objectives:**

This study was performed to investigate the association between depressive symptoms and subclinical thyroid dysfunction.

**Methods:**

Among the 7,550 subjects who participated in the 2014 Korea National Health and Nutrition Examination Survey, 1,763 participants without overt thyroid disease were included in this study. Serum thyroid stimulating hormone (TSH), serum free thyroxine (fT4), and depressive symptoms were analyzed based on the Patient Health Questionnaire (PHQ9).

**Results:**

The percentages of subjects with subclinical hypothyroidism and subclinical hyperthyroidism were 3.3% and 2.6%, respectively. The percentages of subjects with moderate (10–14 points), moderately severe (15–19 points), and severe (≥20 points) depression according to the distribution of PHQ-9 scores were 4.7%, 1.1%, and 0.3%, respectively. TSH, fT4, and the percentage of patients with subclinical hypothyroidism were not significantly associated with PHQ-9 score. However, the percentage of patients with subclinical hyperthyroidism increased significantly with PHQ9 score (P = 0.002). Subjects with subclinical hyperthyroidism had higher PHQ-9 scores than those with normal thyroid function (mean ± standard error [SE], 4.2 ± 0.5 vs. 2.7 ± 0.1 points, P = 0.010). More subjects with subclinical hyperthyroidism had a PHQ9 score ≥ 10 than did those with normal thyroid function (mean ± SE, 17.1 ± 3.5 vs. 5.8 ± 0.6%, P = 0.005). We performed logistic regression analyses for the presence of depressive symptoms, using age, sex, education, household income, alcohol drinking, smoking, diabetes, cerebrovascular disease history, subclinical hypothyroidism, and subclinical hyperthyroidism as variables. Subclinical hyperthyroidism was associated with the presence of clinically relevant depression (PHQ9 score ≥ 10), (odds ratio [OR], 4.04; 95% confidence interval [CI], 1.75–9.31; P = 0.001), and clinically significant depression (PHQ9 score ≥ 15), (OR, 7.05; 95% CI, 1.67–29.67; P = 0.008), respectively. However, subclinical hypothyroidism was not associated with the presence of clinically relevant depression (OR, 1.15; 95% CI, 0.39–3.38; P = 0.800), or clinically significant depression (OR, 3.35; 95% CI, 0.71–15.79; P = 0.127).

**Conclusions:**

We demonstrated that subclinical hyperthyroidism was independently associated with depressive symptoms in the Korean general population using national cross-sectional data.

## Introduction

Thyroid dysfunction is associated with a variety of neuropsychiatric disturbances, including depressive symptoms, mania, acute psychosis, and cognitive disorders [1–3]. In particular, it is well known that untreated hypothyroidism is associated with an increased risk of depression [4]. Actually, thyroid function tests are the most frequently performed endocrine tests for a depression work-up in the primary care clinic [5].

Subclinical hypothyroidism and subclinical hyperthyroidism are usually defined as abnormally high and low serum thyroid stimulating hormone (TSH) levels with a normal serum free thyroxine (fT4) level without clinical signs or symptoms. These disorders are not rare and are even more prevalent than overt thyroid dysfunction. According to data from the 2013–2015 Korea National Health and Nutrition Examination Survey (KNHANES), the prevalence rates of overt and subclinical hypothyroidism are 0.73% and 3.10%, respectively [6]. The prevalence rates of overt hyperthyroidism and subclinical hyperthyroidism are 0.54% and 2.98%, respectively [6].

Depression is one of the most common psychiatric disorders, with substantial morbidity and mortality. Considerable attention has focused on improving the detection and assessment of depression severity. Among many comparable measures for depression, the 9-item Patient Health Questionnaire (PHQ-9), which is a self-report instrument designed to screen depression based on common and internationally valid criteria, is regarded as a reliable and valid measure of depression severity.

In the current study, we performed a cross-sectional analysis to investigate the association between depressive symptoms based on the PHQ-9 and subclinical thyroid dysfunction in a Korean adult population, using data from the 2014 KNHANES.

## Methods

### Study population and data collection

This study was based on data from the 2014 KNHANES, a nation cross-sectional survey conducted by the Korean Center for Disease Control for Health Statistics. As described in detail previously [7], the KNHANES is an independent dataset from the general population of Korea, similar to the National Health and Nutrition Examination Survey in the United States. The health interview includes an established questionnaire to determine the demographic and socioeconomic characteristics of the subjects, including age, education level, occupation, income, marital status, smoking habit, alcohol consumption, exercise, previous and current diseases, and family disease history.

**Fig 1:**
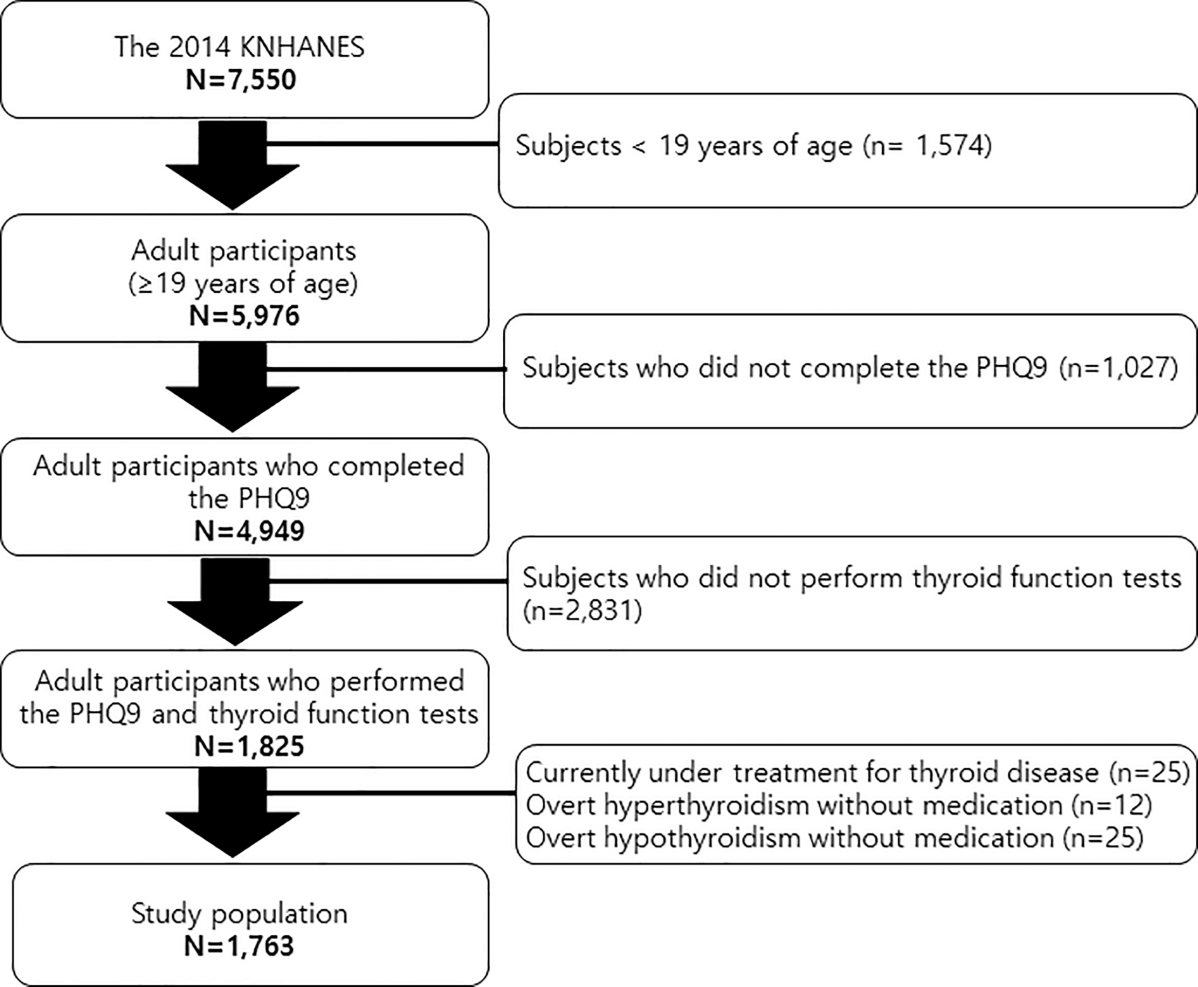
Participants disposition to be included in this study.

Among the 7,550 subjects who participated in the 2014 KNHANES, 1,574 subjects < 19 years of age were excluded. A total of 1,027 adults did not complete the PHQ-9 and were excluded. Among the remaining 4,949 adults, thyroid function tests were performed in 1,825 subjects. (In the 2014 KNHANES, approximately one-third of participants > 10 years, or 2,400 subjects were selected under subsampling considering sociodemographic factors and underwent thyroid function tests. Among the 2,400, 1,825 subjects > 19 years of age completed the PHQ-9. Twenty-five subjects currently under treatment for thyroid disease were excluded. Based on thyroid function tests, 25 subjects with overt hypothyroidism (lower fT4 level than the reference range) and 12 subjects with overt hyperthyroidism (higher fT4 level than the reference range) were also excluded. Finally, 1,763 participants within the normal fT4 range were analyzed in this study (Fig 1).

Diabetes was defined as a fasting plasma glucose (FPG) concentration ≥ 126 mg/dl (7.0 mmol/L), glycosylated hemoglobin (HbA1c) level ≥ 6.5%, current use of antidiabetes medication, or a previous diagnosis of diabetes by a physician.

Systolic and diastolic blood pressures were measured by standard methods using a sphygmomanometer while the patient was seated. Three measurements were recorded for all subjects at 5-min intervals, and the average of the second and third measurements was used for analysis. Hypertension was defined as systolic blood pressure ≥ 140 mmHg, diastolic blood pressure ≥ 90 mmHg, or use of antihypertensive medications regardless of blood pressure.

### Laboratory methods

Blood samples were collected in the morning after at least an 8 h fast. FPG was measured using a Hitachi Automatic Analyzer 7600 (Hitachi, Tokyo, Japan). HbA1c level was measured using high-performance liquid chromatography (HLC-723G7; Tosoh, Tokyo, Japan).

### Assessment of thyroid function

Serum TSH and fT4 concentrations were measured with an electrochemiluminescence immunoassay (Roche Diagnostics, Mannheim, Germany). TSH levels were measured using an E-TSH kit (Roche Diagnostics). We used the 0.62–6.68 mIU/dL Korean reference interval for serum TSH [6], which is higher than that of Western countries. The fT4 was measured using the E-Free T4 kit (Roche Dignostics), and the standard curve range was 0.89–1.76 ng/mL.

### Assessment of depression

Depressive symptoms were assessed using the PHQ-9, which is a questionnaire that can be entirely self-administered by the participant. The PHQ is an instrument for making criteria-based diagnoses of depression and other mental disorders commonly encountered in primary care [8]. The PHQ-9 is the 9-item depression module from the full PHQ. The PHQ-9 has comparable sensitivity and specificity and consists of the actual nine criteria upon which the Diagnostic and Statistical Manual of Mental Disorders Fourth Edition diagnosis of depressive disorders is based [9]. As a severity measure, the PHQ-9 score ranges from 0 to 27, as each of the nine items can be scored from 0 (not at all) to 3 (nearly every day).

PHQ-9 scores of 5, 10, 15, and 20 represent valid and easy-to-remember thresholds demarcating the lower limits of mild, moderate, moderately severe, and severe depression, respectively [9]. A PHQ-9 score ≥ 10 has a sensitivity of 88% and a specificity of 88% for major depression or clinically relevant depression [9]. In particular, PHQ-9 scores ≥ 15 usually signify the presence of clinically significant major depression [9].

### Ethics statement

This study was approved by the institutional review board of Ilsan Paik Hospital, Republic of Korea (IRB 2017-07-035). After approval of the study proposal, the KNHANES dataset was made available at the request of the investigator. Because the dataset does not include any personal information and participants’ consent had already been given for the KNHANES, our study was exempt from participant consent.

### Statistical analyses

Differences in age according to the PHQ-9 score were evaluated by analysis of variance, and the percentage of females was evaluated by the chi-square test. Analysis of covariance and the Bonferroni *post-hoc* test were used to compare the age- and sex-adjusted demographics and clinical characteristics according to the PHQ-9 score.

Analysis of covariance and the Bonferroni *post-hoc* test were also used to compare the age- and sex-adjusted demographics and clinical characteristics according to thyroid function.

A logistic regression analysis was conducted to evaluate the risk of depression using age, sex, education, household income, alcohol drinking, smoking, diabetes, cerebrovascular disease history, subclinical hypothyroidism, and subclinical hyperthyroidism as variables.

All tests were two-sided, and P-values < 0.05 indicated statistical significance. Statistical analyses were performed using SPSS software (ver. 21.0 for Windows; IBM Corp., Armonk, NY, USA).

Power calculations were performed using PASS software (version 12, NCSS, LLC, Kaysville, Utah, USA)

## Results

### Demographics and clinical characteristics of the study population

The demographics and clinical characteristics of the study population are shown in Table 1. Median age was 44 (19–76) years, and 52% of the participants were females. Median fT4 level was 1.24 (0.89–1.76) ng/dL, and median TSH level was 2.23 (0.01–29.50) μIU/dL. The percentages of subjects with subclinical hypothyroidism and subclinical hyperthyroidism were 3.3% and 2.6%, respectively. The median PHQ-9 score was 2 (0–27) points. The percentages of subjects with moderate (10–14 points), moderately severe (15–19 points), and severe (≥20 points) depression according to the distribution of the PHQ-9 scores were 4.7%, 1.1%, and 0.3%, respectively.

**Table 1:** Demographic and clinical characteristics of the study population (n = 1763).

Age (years)	44 (19–76)
Women (%/n)	52.0/917
College graduation (%)	36.2/639
Household income (x10,000 KRW/month)	379 ± 7
Alcohol intake ^3^ 30 g/day (%)	13.8/244
Current smoking (%/n)	24.3/428
Diabetes (%/n)	10.0/177
Hypertension (%/n)	21.9/386
Self-reported coronary heart disease history (%/n)	1.1/19
Self-reported cerebrovascular disease history (%/n)	1.0/18
Self-reported cancer history (%/n)	3.3/58
fT4 (ng/dL)	1.24 (0.89–1.76)
TSH (mIU/mL)	2.23 (0.01–29.50)
Subclinical hypothyroidism (%/n)	3.3/59
Subclinical hyperthyroidism (%/n)	2.6/46
PHQ9 score	2 (0–27)
Level of depression severity,PHQ9 score (%/n)	
Minimal, 0-	78.0/1376
Mild, 5–9	15.8/279
Moderate, 10–14	4.7/83
Moderately severe, 15–19	1.1/19
Severe, 20–27	0.3/6

Data are expressed as mean ± SE or median (range).

### Age, sex, and age- and sex-adjusted clinical characteristics and thyroid function according to the PHQ-9 score

Table 2 shows the age- and sex-adjusted clinical characteristics and thyroid function according to the PHQ-9 score (0–9, 10–14, and ≥ 15). The proportion of women was positively associated with the PHQ9 score (P = 0.001). A significant positive relationship was observed between the PHQ-9 score and alcohol intake (P = 0.009) and cerebrovascular disease history (P < 0.001). A significant negative association was detected between the PHQ-9 score and household income (P < 0.001).

**Table 2:** Age, sex, and age- and sex-adjusted clinical characteristics and thyroid function according to the PHQ9 score.

	PHQ9 score			*P* for difference
	0–9 (n = 1655)	10–14 (n = 83)	15–27 (n = 25)	
Age (years)	44.5 ± 0.4	40.9 ± 1.8	50.1 ± 2.9	0.016
Women (%)	50.9	68.7	72.0	0.001
College graduation (%)	36.8 ± 1.1	28.4 ± 5.1	22.3 ± 9.3	0.086
Household income (x10,000 KRW)	386.1 ± 6.8	297.6 ± 30.6	187.7 ± 55.7	<0.001
Alcohol intake ≥ 30 g/day (%)	13.2 ± 0.8	21.5 ± 3.6	28.0 ± 6.6	0.009
Current smoking (%)	23.6 ± 0.9	35.8 ± 4.2	28.5 ± 7.7	0.017
Diabetes (%)	9.8 ± 0.7	9.6 ± 3.2	25.8 ± 5.8	0.024
Hypertension (%)	21.7 ± 0.9	22.2 ± 4.2	31.7 ± 7.6	0.425
Self-reported coronary heart disease history (%)	1.1 ± 0.3	0.4 ± 1.1	0.0 ± 2.1	0.658
Self-reported cerebrovascular disease history (%)	0.8 ± 0.2	1.5 ± 1.1	15.7 ± 2.0	<0.001
Self-reported cancer history (%)	3.3 ± 0.4	4.1 ± 1.9	2.8 ± 3.5	0.895
fT4 (ng/dL)	1.25 ± 0.01	1.23 ± 0.02	1.22 ± 0.03	0.292
TSH (μIU/mL)	2.71 ± 0.05	2.49 ± 0.23	2.39 ± 0.42	0.519
Subclinical hypothyroidism (%)	3.3 ± 0.4	2.3 ± 2.0	7.4 ± 3.6	0.468
Subclinical hyperthyroidism (%)	2.3 ± 0.4	5.9 ± 1.8	12.1 ± 3.2	0.002
Subclinical hyperthyroidism (%)*	2.3 ± 0.4	6.1 ± 1.8	12.5 ± 3.3	0.001

Data are expressed as mean ± SE.

* Adjusted for age, sex, household income, Alcohol intake ≥ 30 g/day, current smoking, diabetes, and cerebrovascular disease history.

fT4, TSH, and the percentage of subjects with subclinical hypothyroidism were not associated with the PHQ-9 score. However, the percentage of subjects with subclinical hyperthyroidism increased significantly with the PHQ9 score (P = 0.002). After adjusting for age, sex, household income, alcohol intake, current smoking, diabetes, and cerebrovascular disease history, the significance of the association between subclinical hyperthyroidism and PHQ-9 score persisted (P = 0.001).

### Age, sex, and age- and sex-adjusted clinical characteristic and the PHQ-9 score according to thyroid function

Table 3 shows the age, sex, and age- and sex-adjusted clinical characteristics and PHQ-9 scores according to thyroid function. The percentage of subjects with a cancer history was significantly related with subclinical hyperthyroidism (P < 0.001). Subjects with subclinical hyperthyroidism had higher PHQ-9 scores than those with normal thyroid function (mean ± standard error [SE], 4.2 ± 0.5 vs. 2.7 ± 0.1 points, P = 0.010). More subjects with subclinical hyperthyroidism had a PHQ-9 score ≥ 10 points than did those with normal thyroid function (mean ± SE, 17.1 ± 3.5 vs. 5.8 ± 0.6%, P = 0.005). After adjusting for age, sex, and cancer history, the significance of this association between subclinical hyperthyroidism and PHQ-9 score persisted. PHQ-9 score and the percentage of subjects with a cancer history were not associated with subclinical hypothyroidism.

**Table 3:** Age, sex, and age- and sex-adjusted clinical characteristic and the PHQ9 score according to thyroid function.

	Subclinical hypothyroidism (n = 59)		Normal (n = 1658)	Subclinical hyperthyroidism (n = 46)	*P* (Subclinical hypothyroidism vs. normal)	*P* (Subclinical hyperthyroidism vs. normal)
Age (years)		47.9 ± 1.8	44.4 ± 0.4	42.5 ± 2.1	0.219	1.000
Women (%)		64.4	51.4	56.5	0.151	1.000
College graduation (%)		37.8 ± 6.0	36.0 ± 1.1	44.4 ± 6.8	1.000	0.672
Household income (x10,000 KRW)		425.1 ± 36.4	375.6 ± 6.9	446.1 ± 41.2	0.543	0.274
Alcohol intake ≥ 30 g/day (%)		12.6 ± 4.3	13.9 ± 0.8	11.8 ± 4.9	1.000	1.000
Current smoking (%)		20.5 ± 5.0	24.3 ± 0.9	29.6 ± 5.7	1.000	1.000
Diabetes (%)		8.8 ± 3.8	10.1 ± 0.7	9.9 ± 4.3	1.000	1.000
Hypertension (%)						
Self-reported coronary heart disease history (%)		0.0 ± 1.3	1.1 ± 0.3	0.2 ± 1.5	0.972	1.000
Self-reported cerebrovascular disease history (%)		0.0 ± 1.3	1.0 ± 0.2	2.3 ± 1.5	1.000	1.000
Self-reported cancer history (%)		6.0 ± 2.3	2.9 ± 0.4	13.4 ± 2.6	0.545.	<0.001
fT4 (ng/dL)		1.17 ± 0.02	1.25 ± 0.01	1.36 ± 0.02	<0.001	<0.001
TSH (μIU/mL)		10.31 ± 0.20	2.48 ± 0.04	0.38 ± 0.22	<0.001	<0.001
PHQ9 score		3.2 ± 0.5	2.7 ± 0.1	4.2 ± 0.5	0.928	0.010
PHQ9 score*		3.2 ± 0.5	2.7 ± 0.1	4.3 ± 0.5	0.916	0.011
PHQ9 score ≥ 10 (%)		6.4 ± 3.1	5.8 ± 0.6	17.1 ± 3.5	1.000	0.005
PHQ9 score ≥ 10 (%)*		6.4 ± 3.1	5.8 ± 0.6	17.1 ± 3.5	1.000	0.005
PHQ9 score ≥ 15 (%)		3.1 ± 1.5	1.2 ± 0.3	6.5 ± 1.7	0.671	0.008
PHQ9 score ≥ 15 (%)*		3.1 ± 1.5	1.2 ± 0.3	6.6 ± 1.7	0.655	0.007

Data are expressed as mean ± SE.

* Adjusted for age, sex, and cancer history.

### Logistic regression analyses for the presence of depressive symptoms and subclinical thyroid dysfunction

We performed logistic regression analyses for the risk of depression using age, sex, education, household income, alcohol drinking, smoking, diabetes, cerebrovascular disease history, subclinical hypothyroidism, and subclinical hyperthyroidism as variables. Subclinical hyperthyroidism was associated with a risk of clinically relevant depression (odds ratio [OR], 4.04; 95% confidence interval [CI], 1.75–9.31; P = 0.001), and clinically significant depression (OR, 7.05; 95% CI, 1.67–29.67; P = 0.008), respectively (Table 4). The estimated powers were 89 and 75% for clinically relevant depression and clinically significant depression associated with subclinical hyperthyroidism, respectively.

**Table 4:** Odds ratios (ORs) for clinically relevant depression and clinically significant depression.

	Clinically relevant depression		Clinically significant depression	
	(PHQ9 score ≥ 10)		(PHQ9 score ≥ 15)	
Variable	OR (95% CI)	*P*	OR (95% CI)	*P*
Subclinical hyperthyroidism				
No (n = 1717)	Reference (n = 100)		Reference (n = 22)	
Yes (n = 46)	4.04 (1.75–9.31) (n = 8)	0.001	7.05 (1.67–29.67) (n = 3)	0.008
Subclinical hypothyroidism				
No (n = 1704)	Reference (n = 104)		Reference (n = 23)	
Yes (n = 59)	1.15 (0.39–3.38) (n = 4)	0.800	3.35 (0.71–15.79) (n = 2)	0.127

Age, sex, education, household income, alcohol drinking, smoking, diabetes, cerebrovascular disease history, subclinical hypothyroidism, and subclinical hyperthyroidism as variables.

However, subclinical hypothyroidism was not associated with the presence of clinically relevant depression (OR, 1.15; 95% CI, 0.39–3.38; P = 0.800), or clinically significant depression (OR, 3.35; 95% CI, 0.71–15.79; P = 0.127). The estimated powers of our study were 6 and 50% for clinically relevant depression and clinically significant depression associated with subclinical hypothyroidism, respectively.

## Discussion

In this study, subclinical hyperthyroidism, but not subclinical hypothyroidism, was associated with depressive symptoms in the general adult population with normal fT4 levels. In addition, the percentage of subjects and OR of subclinical hyperthyroidism increased significantly along with the severity of depressive symptoms, even after adjusting for several confounding factors.

Previous studies have shown conflicting results about the association between subclinical thyroid dysfunction and depressive symptoms. Some studies have demonstrated that subclinical hypothyroidism increases the risk for depression, particularly in older adults [10,11]. Demartini *et al*. suggested a significant association between subclinical hypothyroidism and depressive symptoms in Italian patients, but most subjects in that study were affected by Hashimoto’s thyroiditis and presented to the department of endocrinology, and were not in the general population [12]. Almeida *et al*. also reported an increased prevalence of depression in Brazilian subjects with subclinical hypothyroidism [13]. However, Park *et al*. showed that subclinical hypothyroidism is not associated with neuropsychiatric derangement, including mood, in older Korean adults [14]. A substudy of the Prospective Study of Pravastatin in the Elderly at Risk (PROSPER) trial also showed that subclinical hypothyroidism is not associated with an increase in depressive symptoms among older Dutch adults at high cardiovascular risk [15]. Because depressive symptoms are affected by various conditions, including cultural and socioeconomic factors, the association between subclinical dysfunction and depression could differ according to the characteristics of the study population, including age, socioeconomic status, and ethnicity. In our study, subclinical hypothyroidism was not associated with the presence of depressive symptoms. However, logistic regression model of subclinical hypothyroidism and depressive symptoms achieved less than 50% power, which might be caused by small number of actual events of depression with subclinical hypothyroidism and low OR. Much larger numbers of subjects are required to replicate this negative result with high power.

Few data show that low TSH within the normal range of thyroid function is associated with depression. Williams *et al*. reported that higher levels of T4 in the normal range are associated with an increased risk of depression using scores from the 30-item General Household Questionnaire in a prospective cohort study of 2,269 middle-aged men [16]. However, that study classified thyroid status only on the basis of total T4 without TSH measures, due to limited availability of a TSH assay at that time. Furthermore, they included only Caucasian males, and the results might not be generalizable to women or other ethnic groups.

Medici *et al*. also identified low-normal TSH as an important risk factor for depression based on the Center for Epidemiologic Studies Depression Scale in 1,503 Dutch men and women [17]. That study was the first to demonstrate a relationship between low-normal TSH levels and depression in both a cross-sectional and longitudinal manner over a mean 8.0-year follow-up. However, that study only included older subjects aged ≥ 55 years and the mean age of the study population was 70.6 years.

Some studies have reported no evidence that subclinical hypothyroidism or subclinical hyperthyroidism contribute to an increased risk of depression [18–20].

All of the above studies were performed mainly in European and United States populations, which are both predominantly Caucasian. The present study is the first nationwide study to show a significant association between subclinical hyperthyroidism and depressive symptoms in a general Asian population.

Although the pathophysiological role of thyroid hormones in depression remains unclear, the relationship between thyroid hormones and the brain serotoninergic (5-HT) system has been suggested as a potential underlying mechanism of action [21]. It is postulated that thyroid hormones regulate cortical 5-HT2 receptor sensitivity and have a modulating impact on the brain serotoninergic system. The hypothalamic-pituitary-thyroid (HPT) axis is also suggested to be involved in the pathogenesis of depression. The most widely recognized disturbance of the thyroid axis, despite normal fT4 levels in depression, is blunting of the TSH response to thyroid releasing hormone (TRH) stimulation [22]. Prolonged release of TRH in subjects with depression may be seen as a compensatory response to decreased 5-HT activity in an attempt to normalize 5-HT function and maintain normal levels of thyroid hormones [23].

There is also another possibility that low TSH does not reflect true thyroid dysfunction but, rather, is a manifestation of secondary effects under depression, underlying systemic medical illness, or non-thyroidal illness syndrome. Although common abnormal findings of thyroid function tests in patients with non-thyroidal illness syndrome are characterized by a low T3 level and normal to low levels of total T4, serum TSH level can also be influenced and suppressed [24]. Actually, a pattern of “euthyroid hyperthyroxinemia” is significantly more common in patients with a mood disorder, whereas elevated TSH levels are highest in patients with substance use disorders [25]. The pathophysiology of changes in thyroid hormone levels under non-thyroidal illness may be mediated by inflammatory cytokines and other mediators, such as interleukin-6 (IL-6), IL-1, and, tumor necrosis factor alpha acting at the level of the HPT axis, as well as tissue deiodinase [26,27].

Several strengths of this study should be mentioned. First, we examined subclinical thyroid dysfunction and depression using national cross-sectional data. Second, because the serum TSH reference range can vary according to geographic and ethnic distribution, we used our own cut-off for normal serum TSH level based on the Korean population, which is higher than that of Western countries. We assumed that iodine intake could be an important factor contributing to the distribution of serum TSH levels in Korea where large amounts of iodine are consumed.

Nevertheless, this study also had some limitations. First, this was not a prospective observational study, and its cross-sectional design prevented us from drawing conclusions regarding causality between low TSH and depressive symptoms. Therefore, we cannot exclude the possibility of a reverse causality. Second, we could not determine without follow-up whether some subjects with low TSH levels might have a form of central hypothyroidism or non-thyroidal illness syndrome. Third, although we excluded participants who were using thyroid medication, we cannot exclude the possibility that our study still included other thyroid interfering medications, such as glucocorticoids, lithium, or amiodarone. Fourth, this study did not evaluate thyroid autoimmunity, which could influence the risk of thyroid dysfunction and even depression [28,29]. Finally, because the PHQ-9 is a self-report screening tool and not a diagnostic tool used by a physician to evaluate depression, the severity of depressive symptoms could have been overestimated or underestimated.

In conclusion, subclinical hyperthyroidism was associated with depressive symptoms in the general Korean population, using national cross-sectional data. Future prospective studies to assess the pathogenic relationship between subclinical hyperthyroidism and depression are needed.
